# Mesenchymal Stem Cell Derived Extracellular Vesicles for Tissue Engineering and Regenerative Medicine Applications

**DOI:** 10.3390/cells9040991

**Published:** 2020-04-16

**Authors:** Dimitrios Tsiapalis, Lorraine O’Driscoll

**Affiliations:** School of Pharmacy and Pharmaceutical Sciences & Trinity Biomedical Sciences Institute, Trinity College Dublin, Dublin, Ireland; tsiapald@tcd.ie

**Keywords:** mesenchymal stem cells, extracellular vesicles, tissue damage, tissue engineering and regeneration, regenerative medicine

## Abstract

Mesenchymal stem cells (MSCs) are being extensively investigated for their potential in tissue engineering and regenerative medicine. However, recent evidence suggests that the beneficial effects of MSCs may be manifest by their released extracellular vesicles (EVs); typically not requiring the administration of MSCs. This evidence, predominantly from pre-clinical in vitro and in vivo studies, suggests that MSC-EVs may exhibit substantial therapeutic properties in many pathophysiological conditions, potentially restoring an extensive range of damaged or diseased tissues and organs. These benefits of MSC EVs are apparently found, regardless of the anatomical or body fluid origin of the MSCs (and include e.g., bone marrow, adipose tissue, umbilical cord, urine, etc). Furthermore, early indications suggest that the favourable effects of MSC-EVs could be further enhanced by modifying the way in which the donor MSCs are cultured (for example, in hypoxic compared to normoxic conditions, in 3D compared to 2D culture formats) and/or if the EVs are subsequently bio-engineered (for example, loaded with specific cargo). So far, few human clinical trials of MSC-EVs have been conducted and questions remain unanswered on whether the heterogeneous population of EVs is beneficial or some specific sub-populations, how best we can culture and scale-up MSC-EV production and isolation for clinical utility, and in what format they should be administered. However, as reviewed here, there is now substantial evidence supporting the use of MSC-EVs in tissue engineering and regenerative medicine and further research to establish how best to exploit this approach for societal and economic benefit is warranted.

## 1. Introduction

For some patients with end-organ dysfunction, whole organ transplantation is an established treatment option [1]. However, the limited availability of suitable autologous tissues, the risk of immune-mediated rejection, the required chronic immunosuppression treatment, and the possibility of disease transmission, highlight the need of new therapeutic approaches [2]. Tissue engineering and regenerative medicine strategies have triggered intense attention due to the potential to develop remedies for damaged, malfunctioning, or injured tissues [3]. Cell-based therapies, in their natural form or modified/engineered for a specific purpose, hold much promise in this regard. Indeed, in light of their multiple sources as well as therapeutic versatility, mesenchymal stem cells (MSCs) have been proposed as the most appropriate cell source for these applications [4,5]. As stem cells, they exhibit beneficial characteristics as compared to terminally differentiated cells, including the potential to circumvent immuno-reaction in vitro and in vivo and to differentiate towards a broad range of specific cell lineages [6,7]. MSCs can be isolated from various tissue types including bone marrow, adipose, umbilical cord, peripheral blood, liver, periodontal ligament, lung and many others [8]. However, despite their potential and promise, MSCs face many challenges, such as their variability, scalability and delivery, as well as ethical considerations and safety issues [9,10,11,12,13], which challenge their clinical utility. 

EVs are heterogeneous lipid bilayer-surrounded vesicles secreted by all cell types, not only MSCs, and act as mediators of intercellular communication. EVs are involved in numerous physiological and pathophysiological biological processes, including modulating immune responses, homeostasis maintenance, coagulation, inflammation, angiogenesis, and cancer progression [14,15,16,17]. According to their size, dimension and origin, they can be classified in many ways, with the preferred terms now being small EVs, medium-sized EVs, and large EVs [18,19]. 

There is increasing evidence that many, if not all, of the beneficial effects of MSCs may be attributed to their paracrine action via the release of extracellular vehicles (EVs), rather than cellular engraftment and response to the site of injury [20,21,22]; suggesting that MSC-EVs can produce any therapeutic benefits of MSCs [23]. Added to their attractiveness, compared to the original MSCs, MSC-EVs cannot self-replicating, preventing safety concerns associated with cell therapy, such as uncontrolled cell division and cellular contamination with tumorigenic cells [24]. Moreover, as MSCs often require invasive procedures in order to be isolated, approaches that only require them to be cultured in vitro and their released product used (i.e., EVs) gives hope for increased scalability and yield per MSC batch [25], with filtration suggested to be suitable sterilisation because of their small size [26]. 

**Figure 1:**
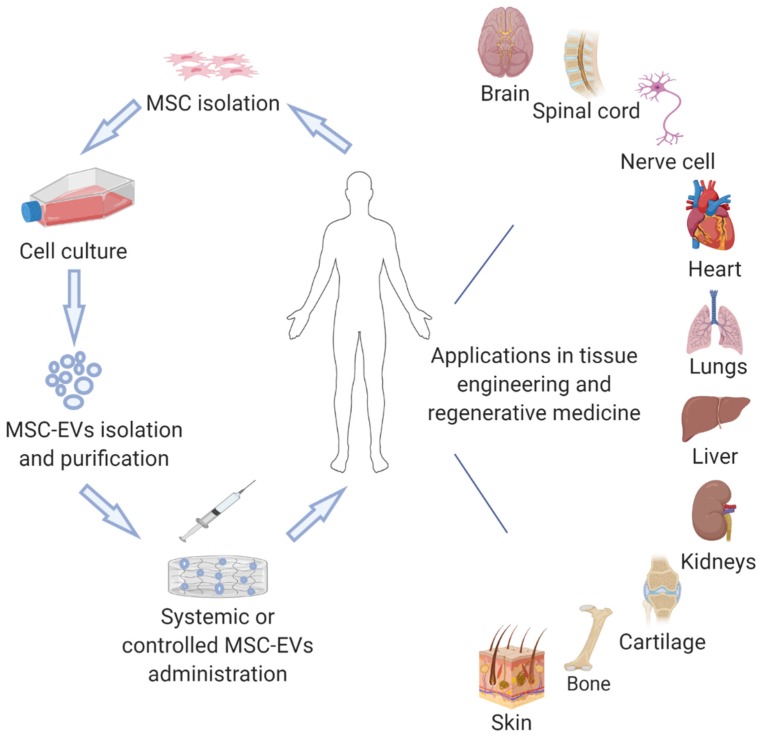
Examples of potential applications of MSC-EVs in tissue engineering and regenerative medicine.

Herein, we review current advancements of the therapeutic potential of MSC-EVs in tissue engineering and regenerative medicine (Figure 1), considering the molecular mechanisms suggested for the MSC-EV action where possible.

## 2. EV Biogenesis and Characterisation 

**Figure 2:**
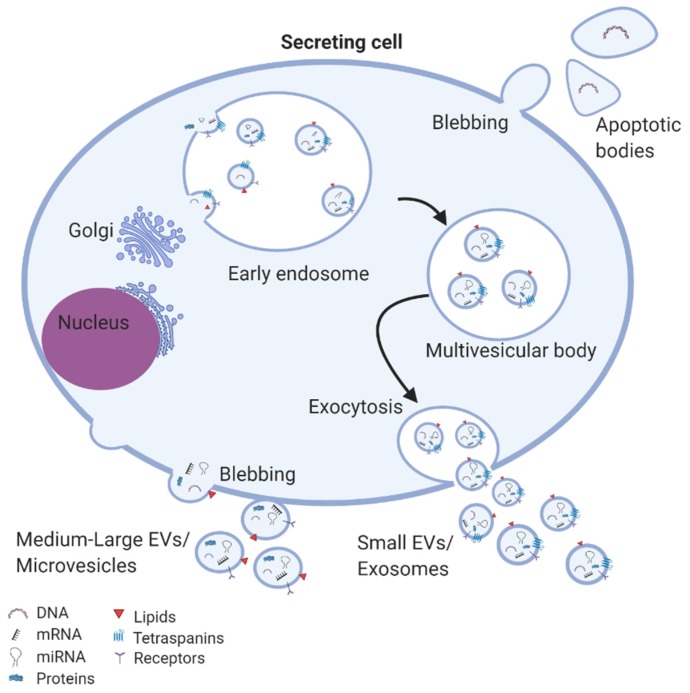
EV biogenesis and secretion: exosomes are assembled in multivesicular bodies where specific cargos are sorted into exosomes and subsequently released in the extracellular space. Microvesicles are formed from budding of the cell membrane. Apoptotic bodies are generated from apoptotic cells.

Small-size EVs, most of which were previously termed as exosomes, are vesicles ranging from 30–150 nm. The biogenesis of the small-size EVs occurs initially with the formation of early endosomes from endocytoses of the cell membrane (Figure 2). During the process of maturation, the early endosomes become endosomes or multivesicular bodies and they begin to accumulate intraluminal vesicles, which either degraded by lysosomes or are released as exosomes in the extracellular space [27]. Medium- and large-size EVs, previously described as microvesicles or ectosomes, are large vesicles between 100–1000 nm diameter. Their biogenesis occurs via the direct budding of the cell membrane and releasing into the extracellular space [28,29]. While apoptotic bodies are also large-size vesicles, ranging from 1–5 mm, they originate specifically from apoptotic cells [30].

As extensively review by the EV research community, according to their cellular origin and the mechanism of secretion, EVs carry somewhat distinct surface markers [21]. Tetraspanin proteins such as CD9, CD81, and CD63 are enriched in the membrane of exosomes and so they are often used to help quality small EVs as exosomes. Furthermore, small EVs may be distinguished by the presence of proteins involved in their biogenesis, including annexin, flotillin, auxiliary proteins (ALIX, TSG101, VPS4), components of the endosomal sorting complex required for transport (ESCRT), GTPase and heat shock proteins (HSP70 and HSP90) [19]. In contrast, CD40 ligand and annexin A1 are associated with medium- and large-size EVs [31,32], whereas apoptotic bodies carry annexin V [33]. Additionally, EV sub-populations contain a range of forms of lipids: cholesterols, diglycerides, sphingolipids (including sphingomyelin and ceramide), phospholipids, and glycerophospholipids, which are key components for the structure, function and biogenesis of EVs [34]. It has been well established that EVs also carry nucleic acids that can be transferred to secondary cells, affecting their cellular processes [17]. Among these, for example, the mRNA content of some EVs has been suggested to significantly influence the biological function of neighbouring cells, such as cell differentiation, transcription, cell proliferation and immune regulation. Similarly, many studies have reported miRNA within EVs being transferred to recipient cells and subsequently altering the gene expression and phenotype of those cells e.g., modulating cycle, apoptosis, migration, inflammation, and neo-angiogenesis [35,36,37,38].

While these studies related to EV biogenesis and characterisation are not all focussed on MSCs, it seems that MSC-EVs are also heterogeneous, carry cargo such as proteins, nucleic acids and lipids, and are involved in cell-to-cell communication.

## 3. EV Isolation Methods

A challenge for MSC-EV in regenerative medicine, particularly when considering towards clinical utility, is the lack of standardisation in EV isolation methods. Of course, this is not unique to MSC-EVs; there are many options but no standardised method for the isolation and purification of EVs. A world-wide survey by the International Society for Extracellular Vesicles (ISEV) [39], considering EV isolation from any and all sourced showed ultracentrifugation-based methods to be most commonly used, although a range of other approaches have been taken to overcome challenges regarding ultracentrifugation including the need for an ultracentrifuge, low-throughput of samples, and potential damage to EVs caused by high-speed centrifugation [40,41]. To this end, alternative methods such as filtration/ultrafiltration, size exclusion chromatography, immunoprecipitation, and precipitation with reagents such as PEG have been utilised, with varying degrees of efficacy in terms of purity and quantity [42]. Thus, combinations of two or more isolation methods are gaining popularity in order to increase EV purity [43]. Moreover, EVs can be modified to enhance their therapeutic potential. Thus, EVs can be incorporated with elements including drugs, antibodies, proteins and RNA for targeted delivery and with molecules such as lipophilic dyes and amino-reactive fluorophores for in vitro and in vivo traceability. In that cases, novel EV-like approaches have been generated such as bio-engineered EVs, EV-mimetic nanovesicles and EV-based semi-synthetic vesicles, as described and reviewed by others [44,45,46,47,48]. Specifically in relation to MSC-EVs, a range of methods of EV isolation from different sources and for different applications to address clinical problems (in the nervous system, heart, bone, cartilage, kidney, liver, muscle, wounds, and other tissues/organs) have been reported, as summarised in Table 1.

## 4. MSC-EVs in Tissue Engineering and Regeneration

### 4.1. Nervous Regeneration

The nervous system is a crucial component of the body and any injury or a disease to it can cause serious or potentially lethal consequences. Nerve repair has long remained a significant objective in regenerative medicine, due to the physiological system complexity and the limited healing capacity [49]. A plethora of strategies have been employed to correct peripheral nerve injuries. In one such study, EVs derived from rat bone marrow mesenchymal stem cells (BMMSCs), were reported to stimulate nerve regeneration after sciatic peripheral nerve crush injury in a rat model. The benefit from the EV was manifest as improved sciatic function index after injury, enhanced histomorphometric repair in nerve regeneration, and increased expression of growth associated protein 43 (GAP43), a marker of axon regeneration [50]. Similarly, peripheral nerve recovery was noticed when EV from human umbilical cord mesenchymal stem cells (UCMSCs) were applied in the site of sciatic nerve defect in rats. EVs aggregated at the site of the nerve injury, prompted the generation of axons and Schwann cells that surrounded individual axons, reduced denervated muscle atrophy and modulated inflammation via down-regulation of pro-inflammatory cytokines (interleukin [IL]-6 and IL-1β) and up-regulation anti-inflammatory cytokines (IL-10) [51]. Another study suggested that EVs from rat adipose-derived mesenchymal stem cells (ADMSCs) promoted peripheral nerve regeneration and neurite growth in sciatic nerve defects, assumed to be through Schwann cell (SC) modulation [52]. Mechanistically, Schwann cells stimulation and proliferation in the damaged neurons relies on the perinuclear location of ADSC-EVs and their accumulation in vesicular-like structures within the Schwann, which indicates an endocytosis-mediated internalization pathway [53]. A more detailed mechanism of peripheral nerve regeneration upon treatment with gingiva MSC-EVs illustrated that the proliferation and migration of Schwann cells occur mainly via the activation of c-Jun N-terminal Kinase (JNK) pathway and the up-regulation of c-Jun, Notch1, glial fibrillary acidic protein (GFAP), and SRY (sex determining region Y)-box 2 (Sox2) characteristic genes of de-differentiation or repair phenotype of Schwann cells [54].

Research efforts showed also the therapeutic efficacy of MSC-EVs in the context of central nervous system (CNS) repair. In a model of middle cerebral artery stroke (MCA), EVs derived from rat BMMSCs led to neurite outgrowth by transfer of miR-133b to neural cells [55]. Systemic administration of rat BMMSC-EVs increased the axonal density and synaptophysin-positive areas along the ischemic boundary zone of the cortex and striatum in MCA rats. This was accompanied by enhanced expression of newly synthesised doublecortin (a marker of neuroblasts [56]) and von Willebrand factor (a marker of endothelial cells [57]) as compared to the untreated controls, suggesting thus neurite remodelling, neurogenesis and angiogenesis as a novel treatment for stroke [58].

With respect to brain damage, rat BMMSC-EVs have been assessed in traumatic brain injury (TBI) rat models. Overall, EV treatment improved recovery of brain function after TBI by increasing the number of newly-generated immature and mature neurons in the dentate gyrus as well as the number of newly-generated endothelial cells in the lesion boundary zone and dentate gyrus [59].

The impact of MSC-EVs has also been investigated in spinal cord injuries (SCI). SCI is frequently associated with microvascular stability disruption and an increase in blood-spinal cord barrier (BSCB) permeability, mainly caused by abnormal migration of pericytes [60,61]. Notably, treatment with mouse BMMSC-EVs inhibited the migration of pericytes and thereby improved the structural integrity of the BSCB and, in turn, the motor function in a SCI rat model [62]. Another potent mechanism of spinal cord recovery upon BMMSC-EVs treatment suggested the prevention of neuronal apoptosis through the activation of the Wnt/β-catenin signalling pathway [63]. Furthermore, in an SCI model, modification of rat BMMSC-EVs with miR-133b caused activation of the ERK1/2, STAT3 pathway. This resulted in enhanced neuron preservation, axon regeneration, and locomotor function, when compared to treatment with BMMSC-EVs that had not been modified to carry miR-133b [64].

MSC-EVs from human placenta have been tested in a multiple sclerosis mouse (MS) model. Here, MSC-EVs induced myelin regeneration in vitro by endogenous oligodendrocyte precursor cells differentiation into mature myelinating oligodendrocytes and increased myelination in the spinal cord of treated mice, following by improved motor function outcomes [65]. Furthermore, EVs from human BMMSCs that had been stimulated with interferon-gamma (IFN-*γ*), reduced neuroinflammation and demyelination improving the motor skills in a MS mouse autoimmune encephalomyelitis (EAE) model [66].

Additionally, in a mouse model of Alzheimer’s disease, MSC-EVs (the source of the MSCs was not detailed) promoted neurogenesis and cognitive function recovery [67].

### 4.2. Cardiac Regeneration

Endogenous myocardial repair after damage is very slow and is dependent on the limited self-division of pre-existing cardiomyocytes and the recruitment and differentiation of resident cardiac stem cells [68,69,70]. The exogenous cell-free approach of using MSC-EVs therapeutically has emerged to address the insufficient responses to myocardial damage typically achievable by endogenous mechanisms. Initial studies established that human embryonic-derived MSC-EVs could reduce infarct size in a mouse model of myocardial ischemia/reperfusion injury (MI) [71], through the activation of the PI3K/Akt signalling pathway, which increased myocardial viability and inhibited adverse remodelling [72]. Taken into consideration this observation, human UCMSCs were subsequently transfected with Akt, which was found to be at high levels in their released EVs. Compared to the non-modified human UCMSC-EVs, this Akt-carrying human UCMSC-EVs complex further accelerated endothelial cell proliferation, migration and tube-like structure formation in vitro and blood vessel formation in vivo [73].

In this setting, some studies have done direct comparisons of MSCs versus MSC-EVs and as a result have highlighted the added beneficial effects of EVs compared to their MSCs of origin. In one such study, murine induced pluripotent mesenchymal stem cells (iPSCs)-EVs and human amniotic fluid-derived mesenchymal stem cells (hAFS) were found to improve cardiac repair in MI murine models and trigger cardiac regeneration via paracrine modulation of endogenous mechanisms. The administered EVs also exhibiting a safer profile when compared to the administration of their cells of origin [74,75]. However, in another MI rat model study, combinatorial treatment with both rat BMMSCs and their derived EVs further improved cardiac function, reduced infarct size, and increased neovascularisation when compared to experimental groups treated with either BMMSCs or BMMSC-EVs alone [76].

Hypoxia pre-conditioning of human BMMSCs has been reported to enhance cells’ biological activities in vitro [77], whilst showed to improve the effectiveness of Cynomolgous monkey BMMSCs when implanted as a treatment of MI in monkeys [78]. Interestingly, hypoxia positively influenced the therapeutic efficacy of the secreted EVs. Hypoxia-elicited human BMMSC-EVs (1% O_2_ for 72 h) showed higher cardiac regeneration in a rat MI model than BMMSCs-EVs isolated under normoxia conditions; the mechanism reported as responsible was by increasing angiogenesis in the site of infract region [79]. Moreover, hypoxia-reconditioning murine and rat BMMSC-EVs (at either 1% O_2_ for 72 h or 0.5% O_2_ for 24 h) prevented cardiomyocyte apoptosis through the enrichment of miR-125b-5p-EVs and miR-210-EVs. The associated mechanism here was suppression of pro-apoptotic genes p53 and BCL2-antagonist/killer 1 (BAK1) and increased recruitment of cardiac progenitor cells in the infarcted heart [80,81].

As an alternative approach to injecting MSC-EVs to the cardiac defect, they can be encapsulated to hydrogels for controlled and targeted administration. Hence, sustain release profile and increased cardiac regeneration was noticed when human UCMSC-EVs were loaded in functional peptide hydrogels. Specifically, the EV/hydrogel complex improved the myocardial function by reducing inflammation, fibrosis and apoptosis, and by promoting angiogenesis in infarcted border zone of rat hearts [82].

### 4.3. Bone Regeneration

The paracrine effects of MSCs —through the use of their EVs— also offer a potential alternative approach for skeletal regeneration. To this end, EVs from different sources of MSCs are being investigated for bone reconstruction after injury. It was noted that human BMMSC-EVs and human iPSCs-EVs stimulated osteogenic differentiation of BMMSCs in vitro, and bone formation and angiogenesis BMMSCs in vivo in rat models with critical-sized calvarial defects [83,84]. To enhance the bone healing, the human BMMSC-EVs were modified with dimethyloxaloylglycin, which resulted in further stimulation of angiogenesis through the Akt/mTOR pathway [85]. The efficacy of EVs derived from human ADMSCs towards bone regeneration was also enhanced by pre-conditioning the MSCs with the cytokine tumour necrosis factor-alpha (TNF-α), as was evidenced by increased proliferation and osteogenic differentiation of osteoblastic cells in vitro [86]. Furthermore, in a femoral shaft fracture model of CD9^−/−^ mice whose bone healing capacity is impaired compared to that of wild-type equivalents, administration of human BMMSC-EVs rescued the delay in fracture healing, and accelerated bone repair in wild types [87]. 

For dental regeneration, the addition of EVs derived from human dental pulp MSCs resulted in odontogenic differentiation in vitro as a results of endocytosis of the EVs, subsequent activation of the P38 mitogen activated protein kinase (MAPK) pathway, and regeneration of dental pulp-like tissue in a tooth root slice model [88]. Moreover, MSC-EVs promoted periodontal ligament cell migration and proliferation through CD73-mediated adenosine receptor activation of pro-survival Akt and ERK signalling and periodontal regeneration in a rat periodontal defect model [89].

With the aim of augmenting scaffold performance and thereby improving bone healing, tissue engineered-constructs have been combined with MSC-EVs. Thus, human ADMSC-EVs were immobilised to poly (lactic-co-glycolic acid) and biotin-doped polypyrrole titanium scaffolds. In vitro studies of scaffolds functionalised with the EVs versus unmodified scaffolds showed the former to results in higher osteo-inductivity of BMMSCs and osteoblasts, while in vivo studies using murine models of bone defects showed significantly greater bone tissue and mature collagen formation [90,91]. Additionally, human BMMSC-EVs loaded into tricalcium phosphate scaffolds enhanced bone healing of calvarial defects by activation PI3K/Akt signalling pathway [92], whilst rat BMMSC-EVs encapsulated into decalcified bone matrix scaffolds stimulated bone regeneration by promoting vascularisation in the grafts [93]. Moreover, three-dimensional polylactic acid scaffolds functionalised with human gingival MSCs have been used as a therapeutic tool for bone tissue engineering, with promising osteogenic properties and response in rat models of cortical calvaria bone damage [94]. 

### 4.4. Cartilage Regeneration

Articular cartilage has limited intrinsic regenerative capacity upon injury and if poorly healed may lead to osteoarthritis (OA); a severe disease accompanied with loss of joint function and devastating pain [95]. MSC-EVs from various cell sources provide new insights for the development of cell-free therapies for the treatment of cartilage injuries and OA. The therapeutic efficacy of MSC-EVs over their cells of origin for the treatment of OA was highlighted in a comparison study using amniotic fluid MSCs and their derived EVs. EV-treated defects showed superior pain tolerance level and improved histological scores than the MSC-treated defects [96]. Furthermore, human BMMSC-EVs have been reported to promote in vitro cartilage regeneration by triggering the production of collagen type II and proteoglycans of chondrocytes isolated from OA patients [97]; both of which are extracellular matrix (ECM) components essential for the proper cartilage repair [98]. It is widely accepted that OA is associated with cartilage degradation mediated mainly by Wnt5A, a non-canonical Wnt protein, which can activate matrix metalloproteinases (MMPs) and reduce the formation of cartilage ECM [99]. Human BMMSC-EVs modified to be enriched with miR-92a-3p have been reported to suppress cartilage degradation and promote cartilage repair in vitro and in an in vivo OA mouse model, as a result of miR-92a-3p targeting Wnt5A [100]. Furthermore, pre-conditioning of rat MSCs with transforming growth factor beta (TGFβ) enhanced the quantities of miR-135b in the resulting EVs, which stimulated chondrocyte proliferation in vitro through specificity protein 1 (Sp1) regulation and cartilage tissue repair in a rat model of OA [101].

In studies applying human embryonic MSC-EVs in rat and mouse models with osteochondral defects, it was noted that osteochondral regeneration was mediated through distinct well-orchestrated mechanisms, such as by enhancing chondrocyte proliferation; attenuating apoptosis; and regulating immune reactivity in the site of the injury; by balancing the synthesis and degradation of cartilage ECM and by restoring matrix homeostasis [102,103,104,105].

In addition to enriching EVs with specific miRNA as summarised above, other strategies have been used to augment the potency of MSC-EVs in cartilage repair. For instance, three-dimensional culture of UCMSCs in a hollow-fibre bioreactor resulted in a higher yield of EVs and superior therapeutic efficacy in a rabbit cartilage defect model, compared to MSC-EVs from conventional 2D cultures [106]. To ensure that MSC-EVs are retained at the site cartilage injury, human iPSC-EVs have been incorporated with in situ hydrogel glue. This acellular tissue patch was found to integrate with native cartilage matrix and promote cell deposition at cartilage defect sites, resulting in functional cartilage repair [107]. 3D printing has been described as the next generation of fabrication techniques in tissue engineering that enable the development of complex forms with high precision [108]. Interestingly, BMMSC-EVs together with cartilage ECM/gelatin methacrylate hydrogel have been used as bio-inks for the design of a bio-scaffold. The resulting 3D printed device promoted the targeted delivery of EVs, preventing mitochondrial dysfunction in degenerative chondrocytes in vitro, and facilitated the cartilage regeneration in an in vivo osteochondral defect rabbit model [109].

### 4.5. Kidney Regeneration

The number of studies using MSC-EVs for the treatment of acute kidney injuries (AKI) and chronic kidney damage (CDK) is continuously increasing, suggesting this strategy may be a promising approach for kidney regeneration. Initially it was established that human BMMSC-EVs accelerated the recovery of injured tubular cells, stimulated cell proliferation, prevented apoptosis and supported functional recovery of glycerol-induced AKI [110]. The suggested route of MSC-EV action involved the delivery of genetic material—such as mRNAs and miRNAs—to injured renal cells, contributing to anti-inflammatory, anti-apoptotic, anti-fibrotic, and pro-angiogenesis effects on AKI [111,112]. Another reported mechanism of renal repair in response to BMMSC-EVs treatment includes the horizontal transfer of human IGF-1 receptor mRNA, which is present in MSC-EVs, to tubular cells [113]. Moreover, administration of human and mice BMMSC-EVs in rat and mice AKI models, respectively, protected the animals from AKI and improved renal function; by inhibiting apoptosis and stimulating tubular epithelial cell proliferation [114,115]. In a cisplatin-induced toxic AKI mouse model, human BMMSC-EVs ameliorated renal function and morphology, and improved survival. Considering the mechanism responsible for this in vitro in cisplatin-treated human tubular epithelial cells, it was shown that the EVs up-regulated anti-apoptotic genes (B-cell lymphoma extra-large B-cell lymphoma 2 and baculoviral IAP repeat containing 8) and down-regulated genes that contribute in the execution-phase of cell apoptosis (caspase-1, caspase-8 and lymphotoxin alpha) [116]. A significant better EV efficacy followed by improved renal function was noticed when mice BMMSC-EVs were loaded to self-assembling peptide nanofiber hydrogels, for control and targeted release of EVs on the site of mice AKI models after ischaemia-reperfusion [117]. Aside from bone marrow, MSC-EVs from other tissues have been isolated and evaluated for renal regeneration. In one such study, human UCMSC-EVs induced in vitro and in vivo renal repair in rat AKI models (cisplatin-induced and unilateral), through reducing oxidative stress and cell apoptosis, promoting cell proliferation, and tubular cells de-differentiation [118,119]. Similar renal regeneration was observed when EVs derived from human Wharton’s Jelly MSCs were applied in AKI rat models. It was also reported that EVs improved renal function by enhancing tubular cell proliferation and reducing inflammation and apoptosis via mitochondrial fission [120,121]. EVs isolated from human glomerular MSCs and liver MSCs have also been credited with stimulating recovery after AKI [122,123].

Several studies have assessed the influence of MSC-EVs in models of chronic kidney damage (CDK) mainly caused by diabetes [124]. Human urinary MSC-EVs have been reported to prevent CKD progression, by inhibiting podocyte apoptosis and promoting vascular regeneration and cell survival in a rat model of streptozotocin-induced diabetic nephropathy [125]. Another study revealed an improvement of renal morphology with profound anti-apoptotic behaviour of tubular epithelial cells when urinary MSC-EVs were injected into diabetic mice [126]. Human BMMSC-EVs and human liver MSC-EVs inhibited fibrosis and prevented its progression in a mouse model of diabetic nephropathy mediated by miRNAs capable of down-regulating profibrotic genes [127]. Analogous observations were noticed upon administration of human liver MSC-EVs in a CKD model induced by aristolochic acid [128]. Murine BMMSC-EVs protected against renal injury both in vitro and in vivo by microRNA-dependent repair in CKD mice models of surgical 5/6 nephrectomy of the kidney tissue [129,130]. Furthermore, injection of human BMMSC-EVs repaired the damage to apical and basolateral membranes and mitochondria of kidney proximal tubules, and improved renal function in a cadmium medaka model resembling CKD due to long-term environmental exposure to heavy metal [131]. In a clinical trial in forty CKD patients stage III and IV (*n* = 20 administered MSC-EVs, *n* = 20 administered placebo) it was observed that MSC-EVs derived from umbilical cord are safe and were able to ameliorate the progression of CDK in grade III-IV CKD patients [132].

### 4.6. Liver Regeneration

Evaluating the potential benefits of MSC-EVs in relation to liver disease, in a carbon tetrachloride (CCl4)-induced liver injury mouse model human embryonic MSC-EVs were found to promote hepatic regeneration, by increasing hepatocyte proliferation and reduced hepatocyte apoptosis [133]. Moreover, human iPSC-EVs enhanced hepatic regeneration in hepatic ischemia-reperfusion injury rat models, by inhibiting apoptosis of hepatic cells, suppressing inflammatory responses, and attenuating the oxidative stress response [134]. Human iPSC-EVs were also reported to induce hepatocyte proliferation in vitro and in vivo in a dose-dependent manner, which is related to the activation of sphingosine kinase and sphingosine-1-phosphate signalling pathway [135], known to promote cell proliferation in various cell types [136,137,138]. Similarly, treatment with human UCMSC-EVs has been shown to ameliorate the infiltration of neutrophils and diminish oxidative stress in hepatic tissue; therefore protecting against hepatic apoptosis [139]. To further enhance the benefits of EVs, human embryonic MSC-EVs were encapsulated in PEG hydrogels for sustain systemic delivery against hepatic failure. Here, EVs accumulated in the liver of the rat model of chronic hepatic fibrosis for prolonged time, exerting superior anti-apoptosis, anti-fibrosis and regenerative properties as compared to conventional EV injection [140].

### 4.7. Muscle Regeneration

The influence of MSC-EVs have been also assessed in skeletal muscle regeneration. For example, human BMMSC-EVs were found to augment myogenesis and angiogenesis in vitro (mediated by miRNAs such as miR-494) and to enhanced muscle regeneration [141]. Moreover, it was noted that EVs derived from amniotic fluid MSCs contain a spectrum of proteins and miRNAs capable of regulating inflammation and angiogenesis which, in turn, underpin skeletal muscle regeneration [142]. Bioinformatic (miRNA profile and proteomics) analysis of a study assessing the regenerative effect of human ADMSC-EVs on muscle injury showed that repair was mediated by factors distributed both within MSC-EVs and the soluble fraction of the secretome [143].

As a preventative measure, EVs isolated from human ADMSCs have been tested as a means to prevent muscle injuries related to torn rotator cuffs. Here, MSC-EV treatment prevented the atrophy, fatty infiltration, inflammation, and vascularisation of muscles in a rat model of torn rotator cuffs and, also, increased the myofiber regeneration and biomechanical properties of the muscles in rotator cuffs [144]. Furthermore, human urine-derived MSC-EVs promoted repair of pubococcygeus muscle injury in rat models of stress urinary incontinence, through stimulating phosphorylation of extracellular-regulated protein kinases and the activation, proliferation, and differentiation of muscle satellite cells [145]. Additionally, human ASC-EVs have recently been shown to prevent muscle damage in a mouse model of critical hindlimb ischemia, mainly through neuregulin 1 protein (NRG1)-mediated signals playing a crucial role in angiogenesis, prevention of inflammation, and muscle protection [146].

### 4.8. Wound Healing

Wound healing is a dynamic process that requires a complex of molecular and cellular events, including cellular migration, proliferation, angiogenesis, ECM deposition, and tissue remodelling [147]. Wounds that exhibit impaired or improper healing have failed to progress through the normal stages of healing i.e., homeostasis, inflammation, proliferation, and remodelling; leading to the formation of excessive scars [148]. Several studies have demonstrated the beneficial activities of MSC-EVs for various chronic wounds. In one such study, BMMSC-EVs enhanced, in a dose-dependent manner, the ex vivo proliferation and migration of fibroblasts from healthy donors and chronic wound patients. These EVs also mediated tube formation by endothelial cells, through the activation of pathways (Akt, ERK, and STAT3) involved in wound healing [149]. Another in vitro study suggested that cutaneous wound healing could be facilitated by increasing collagen synthesis and angiogenesis following treatment with human iPSC-EVs [150]. Higher collagen and elastin synthesis was also noted when human ADMSC-EVs were added to photo-damaged human dermal fibroblasts in vitro [151]. In vivo using a mouse skin incision model, injection of human ADMSC-EVs accelerated wound healing via modifying the phenotypic characteristics of fibroblasts. Specifically, collagen I and III distributions secreted by fibroblasts were increased in the early stage of wound healing while, in the late stage, collagen synthesis was diminished to reduce scar formation [152]. EVs from the same source were reported to trigger the migration and proliferation of keratinocytes and fibroblasts in vitro and in vivo in excisional wound-splinting rat models, by a mechanism involving the activation of Akt pathway [153]. lncRNA metastasis-associated lung adenocarcinoma transcript 1 found in human ADMSC-EVs contributed significantly to the migration and proliferation of dermal fibroblasts, promoting wound closure in a rat model of ischemic wound healing [154]. In a comparison study examining the effect of rabbit ADMSC-EVs and BMMSC-EVs on rat cutaneous wound models, treatment with ADMSC-EVs showed significant better healing [155]. 

Moreover, human UCMSC-EVs have been reported to intensively promote the healing of second-degree burn wounds in vivo, mediated mainly by the activation of Wnt/β-catenin signalling pathway and subsequent increased dermal fibroblasts proliferation, angiogenesis, and reduced skin cell apoptosis [156,157,158]. Wound healing and suppressed scar formation have been facilitated by inhibiting myofibroblast differentiation at the site of skin defects by treating with human UCMSC-EVs. This therapeutic benefit of EVs has been particularly credited to the activities of specific microRNAs (namely, miR-21, -23a, -125b, and -145) [159].

### 4.9. The Regenerative Effect of MSC-EVs on Various Tissues

Aside from their reported beneficial therapeutic effect on the mentioned tissues and organs, MSC-EVs have been examined for their ability to restore or, indeed, improve the performance of many other organs and body systems -such as lungs, blood vessels, oesophagus and bowel- in case of injuries or diseases. For instance, it was reported that human placenta MSC-EVs could attenuate injuries (upon lipopolysaccharide stimulation) caused in lung cells in vitro [160]. In vivo, swine BMMSC-EVs improved lung function in a pig model of influenza virus-induced acute lung injury [161]. Furthermore, human BMMSC-EVs have been shown to alleviate pulmonary vascular permeability and lung injury induced by haemorrhagic shock and trauma in a mouse model, through the activation of proteins and pathways linked to cytoskeletal rearrangement of vascular permeability [162].

Additionally, human placenta MSC-EVs have been shown to inhibit calcification of synthetic vascular grafts by immunomodulation and improving vascular performance and functionality and in a rat model of hyperlipidaemia [163]. Likewise, EVs isolated from ADMSCs limited the abnormal proliferation and migration of vascular smooth muscle cells, followed by neointimal hyperplasia in the setting of vein graft bypass surgery [164]. Administration of human UCMSC-EVs in a mouse model of hind-limb ischemia ameliorated severe ischemic injury, as revealed by increased limb perfusion and function [165]. With respect to tracheoesophageal diseases such as fistulas, ADMSC-EVs embedded in thermo-responsive hydrogels ensured the targeted delivery of EVs at the site of porcine oesophageal fistula; this, in turn, augmented healing. The proposed mechanism here involved the inhibition of myofibroblast proliferation and fibrosis, the decline of inflammatory response, and the enhancement of angiogenesis [166]. Finally, administration of mouse BMMSC-EVs improved the symptoms of ulcerative colitis in a dextran sodium sulfate-induced mouse model, by stimulating M2 macrophage polarisation and blocking inflammatory response [167]. 

## 5. Conclusions and Future Perspectives

As reviewed here, due to their intrinsic therapeutic capacity EVs derived from MSCs—of a range of origins— represent a powerful tool for the treatment of many injuries and diseases, opening multiple novel avenues for tissue engineering and regenerative medicine strategies. While the number of clinical studies is limited to date (Table 2), there is evidence to show that the beneficial effects of MSC-EVs could be further enhanced by bioengineering and genetic modification; stimulation with a variety of different biophysical and biochemical stimuli; drug encapsulation; and nanomaterial science.

However, there are still challenges to the development of MSC-EVs for clinical use. From a practical point-of-view, a major challenge is establishing the optimal reliably, reproducible and robust methodologies for isolation and purification of the therapeutic EVs and their large-scale production at cGMP standard for clinical utility. Moreover, maybe yet from a more fundamental research point-of-view is to establish which sub-populations (if not all) of the heterogenous EV populations are therapeutically beneficially. The clear classification into different subtypes is still under investigation. Further research is also warranted to establish suitable therapeutic doses and optimal route(s) of administration for clinical utility in the future.

## Figures and Tables

**Table 1:** MSC-EVs in tissue engineering and regenerative medicine applications.

Injury/Damage	Cell Source	Isolation Method	Administration Route/Quantity	Main Findings from Studies Evaluating These EVs
**Nervous System**				
Sciatic peripheral nerve crush	Rat BMMSCs	Ultracentrifugation (100,000× *g*)	Injection/45 μg total EV protein in 30 μL PBS	Improved sciatic function index, enhanced histomorphometric repair in nerve regeneration and increased GAP-43 expression [50]
Sciatic peripheral nerve crush	Human UCMSCs	Differential centrifugation and ultracentrifugation (100,000× *g*)	Injection/100 μg total EV protein	Generation of axons and Schwann cells, reduction of denervated muscle atrophy and modulation of inflammation [51]
Sciatic peripheral nerve crush	Rat ADMSCs	Differential centrifugation and ultracentrifugation (100,000× *g*)	Injection/details not provided	Peripheral nerve regeneration and neurite growth in sciatic nerve defects through Schwann cell (SC) modulation [52]
Peripheral nerve injury (in vitro)	Rat ADMSCs	Total Exosome isolation reagent kit (Invitrogen)	2 and 8 µg total EV protein	Enhanced proliferation of Schwann cells in the site of peripheral nerve injuries via internalisation [53]
Sciatic peripheral nerve crush	Human gingiva MSCs	ExoQuick-TC kit (System Biosciences)	EV-scaffold transplantation/40 μg total EV protein in 20 μL PBS	Enhanced proliferation and migration of Schwann cells via the activation JNK pathway and the up-regulation of c-JUN, Notch1, GFAP and SOX2 [54]
Cerebral artery stroke (in vitro)	Rat BMMSCs	Sucrose gradient ultracentrifugation (100,000× *g*)	Details not provided	Neurite outgrowth by transfer of miR-133b to neural cells [55]
Cerebral artery stroke	Rat BMMSCs	Sucrose gradient ultracentrifugation (100,000× *g*)	Injection/100 μg total EV protein in 500 μL PBS	Increased axonal density and synaptophysin-positive areas along the ischemic boundary zone of the cortex and striatum [58]
TBI	Rat BMMSCs	ExoQuick-TC kit (System Biosciences)	Injection/100 μg total EV protein in 500 μL PBS	Improved recovery of brain function by increasing the number of number of neurons and endothelial cells in the lesion boundary zone and dentate gyrus [59]
SCI	Mouse BMMSCs	Differential centrifugation and sequential ultracentrifugation (up to 110,000× *g*)	Injection/200 μL derived from 1 × 10^6^ MSCs post-SCI and 200 μL derived from 1 × 10^6^ MSCs at 1-day post-injury	Reduced migration of pericytes and improved structural integrity of the BSCB [62]
SCI	Rat BMMSCs	Differential centrifugation and ultracentrifugation (100,000× *g*)	Injection/200 μg total EV protein in 200 μL PBS	Reduced neuronal apoptosis through the activation of the Wnt/β-catenin signalling pathway [63]
SCI	Rat BMMSCs	ExoQuick-TC kit (System Biosciences)	Injection/100 μg total EV protein in 500 μL PBS	Modification of rat BMMSC-EVs with miR-133b activated the ERK1/2, STAT3 pathway, which resulted to enhanced neuron preservation [64]
MS	Human placental MSCs	Differential centrifugation and ultracentrifugation (112,700× *g*)	Injection/1 × 10^7^ or 1 × 10^10^ particles in 200 μL PBS	Increased myelination in the spinal cord of treated mice and improved motor function [65]
EAE	Human BMMSCs	Differential centrifugation and ultracentrifugation (120,000× *g*)	Injection/150 μg of total EV protein	Reduced neuroinflammation, demyelination and improved motor function
Alzheimer’s disease	Details not included	Differential centrifugation and ultracentrifugation (110,000× *g*)	Injection/10 µg total EV protein in 2 µL PBS	Enhanced neurogenesis and cognitive function recovery [67]
**Cardiac**				
MI	Human embryonic MSCs	Sucrose gradient ultracentrifugation (200,000× *g*)	Injection/3 µg total EV protein in 200 µL PBS	Reduced infarct size [71]
MI	Human embryonic MSCs	Tangential flow filtration	Infusion/0.4 μg total EV protein in 1 mL PBS	Increased myocardial viability and inhibited adverse remodelling [72]
MI	Human UCMSCs	Sucrose gradient ultracentrifugation (100,000× *g*)	Infusion/400 µg of total EV protein	Akt-modified MSC-EVs promoted endothelial cell proliferation, migration, tube-like structure formation, and blood vessel formation [73]
MI	Murine iPSCs	Differential centrifugation and ultracentrifugation (110,000× *g*)	Injection/100 µg of total EV protein in 30 μL PBS	Increased cardiac repair, left ventricular function, vascularization, and reduced apoptosis and hypertrophy [74]
MI	Human amniotic fluid-derived MSCs	Serial ultracentrifugation	Injection/4.5 μg total EV protein	Improved cardiac regeneration via paracrine modulation of endogenous mechanisms [75]
MI	Rat BMMSCs	Differential centrifugation and ultracentrifugation (120,000× *g*)	Injection/10 μg total EV protein in 100 μL PBS	BMMSCs and their derived EVs synergistically improved cardiac function, reduced infarct size, and increased neovascularization [76]
MI	Human BMMSCs	Ultracentrifugation (100,000× *g*)	Injection/80 μg total EV protein	Hypoxia-elicited BMMSC-EVs showed higher cardiac regeneration as compared to MSC-EVs isolated in normoxia [79]
MI	Murine BMMSCs	Differential centrifugation and ultracentrifugation (140,000× *g*)	Injection/200 µg of total EV protein in 200 μL PBS	Hypoxia-elicited BMMSC-EVs prevented cardiomyocyte apoptosis through the enrichment of miR-125b-5p [80]
MI	Rat BMMSCs	Repeated ultracentrifugation	Injection/EVs derived from 2 × 10^7^ MSCs, in 30 μL PBS	Hypoxia-elicited BMMSC-EVs enhanced the cardioprotective actions of EVs [81]
MI	Human UCMSCs	Total exosome isolation kit (Life Technologies)	EV-hydrogel transplantation/20 µg of total EV protein in 20 μL PBS	Sustain delivery of MSC-EVs improved myocardial function by reducing inflammation, fibrosis and apoptosis, and by promoting angiogenesis [82]
**Bone**				
Critical-sized calvarial defect	Human BMMSCs	Differential centrifugation and ultracentrifugation (120,000× *g*)	EV-hydrogel transplantation/100 μg of total EV protein in 50 μl PBS	Increased angiogenesis and bone formation [83]
Critical-sized calvarial defect	Human iPSCs	Ultrafiltration and gradient ultracentrifugation (100,000× *g*)	EV-scaffold transplantation/100 μg or 200 μg of total EV protein	Increased angiogenesis, osteogenesis and bone formation [84]
Critical-sized calvarial defect	Human BMMSCs	Differential centrifugation and ultracentrifugation (110,000× *g*)	Injection to implanted scaffolds/100 μg of total EV protein in 200 μL PBS	BMMSC-EVs modified with dimethyloxaloylglycin enhanced bone regeneration through Akt/mTOR pathway activation and angiogenesis stimulation [85]
Bone regeneration (in vitro)	Human ADMSCs	Ultracentrifugation (100,000× *g*)	Details not provided	TNF*α*-primed ADMSC-EVs enhanced proliferation and differentiation of osteoblastic cells by increasing the expression of Wnt-3a [86]
Femoral shaft fracture(CD9^−/−^ and wild types)	Human BMMSCs	Ultracentrifugation (180,000× *g*)	Injection / details not provided	Improved fracture healing in CD9^−/−^ mice, and accelerated bone repair in wild types [87]
Tooth root slice	Human dental pulp MSCs	ExoQuick-TC kit (System Biosciences)	Injection/details not provided	Increased odontogenic differentiation regeneration of dental pulp-like tissue [88]
Periodontal defect	Unknown	Tangential flow filtration	EV-scaffold transplantation/40 μg of total EV protein	Enhanced periodontal ligament cell migration, proliferation and periodontal regeneration [89]
Critical-sized skull defect	Human ADMSCs	Differential centrifugation and ultracentrifugation (100,000× *g*)	EV-scaffold transplantation/1 μg of total EV protein in 1 μL PBS	MSC-EVs immobilised in poly(lactic-co-glycolic acid) stimulated the controlled release of EVs promoting MSC migration and homing in the bone defects [90]
Ectopic bone formation	Human ADMSCs	Differential centrifugation	EV-scaffold transplantation/10 μg of total EV protein in 1 mL PBS	MSC-EV immobilised constructs showed higher osteo-inductive ability and long-term stability for bone graft modification [91]
Critical-sized calvarial defects	Human BMMSCs	Differential centrifugation and sucrose gradient ultracentrifugation (100,000× *g*)	EV-scaffold transplantation/5 × 10^11^ or 1 × 10^12^ particles in 1 mL PBS	BMMSC-EVs loaded into tricalcium phosphate scaffolds promoted osteogenesis activity and bone regeneration [92]
Ectopic bone formation	Rat BMMSCs	Differential centrifugation	EV-scaffold transplantation/20 μg of total EV protein in 20 μL PBS	MSC-EVs loaded into decalcified bone matrix scaffolds stimulated neo-vascularization and bone formation [93]
Cortical calvaria bone defect	Human gingival MSCs	ExoQuick-TC kit (System Biosciences)	EV-scaffold transplantation/details not provided	MSC-EVs loaded in three-dimensional polylactic acid scaffolds enhanced osteogenic properties and improved bone healing [94]
**Cartilage**				
OA	Human amniotic fluid MSCs	Total exosome isolation kit (Life Technologies)	Injection/100 μg of total EV protein	EV-treated defects showed superior pain tolerance level and improved histological scores than the MSC-treated defects [96]
Cartilage regeneration (in vitro)	Human BMMSCs	Differential centrifugation and ultracentrifugation (100,000× *g*)	Details not provided	Increased production of collagen type II and proteoglycans in chondrocytes isolated from OA patients [97]
OA	Human BMMSCs	Differential centrifugation and sucrose gradient ultracentrifugation (100,000× *g*)	Injection/500 μg of total EV protein in 1 mL PBS	BMMSC-EVs modified with miR-92a-3p, suppressed cartilage degradation by targeting the WNT5A and promoted cartilage repair [100]
OA	Rat MSCs	Tangential flow filtration	Injection/1 × 10^11^ particles in 1 mL PBS	TGF*β* primed MSC-EVs promoted cartilage tissue repair through Sp1 regulation [101]
OA	Human embryonic MSCs	Tangential flow filtration	Injection/100 µg of total EV protein in 100 µL PBS	Increased chondrocyte proliferation, reduced apoptosis, regulated inflammation and matrix homeostasis [102,103,104]
OA	Human embryonic MSCs	Differential centrifugation and ultracentrifugation (100,000× *g*)	Injection/details not provided	Improved osteoarthritis through balancing the synthesis and degradation of cartilage ECM [105]
Cartilage defect	Human UCMSCs	Differential centrifugation and ultracentrifugation (110,000× *g*)	Injection/1 × 10^10^ particles in 1 mL PBS	UCMSCs cultured in a bioreactor resulted in a higher yield of EVs and superior therapeutic efficiency [106]
Articular cartilage defect	Human iPSCs	Differential centrifugation, ultracentrifugation (100,000× *g*) and ultrafiltration	EV-scaffold transplantation/1 × 10^11^ particles in 1 mL PBS	iPSC-EVs incorporated with in situ hydrogel glue could integrate with native cartilage matrix and promote cell deposition at cartilage defect [107]
Osteochondral defect	BMMSCs	Ultrafiltration and sucrose gradient ultracentrifugation (100,000× *g*)	EV-scaffold transplantation/200 µg of total EV protein in 200 µL PBS	BMMSC-EVs together with cartilage ECM/ gelatin methacrylate hydrogel have been used to create a 3D printed device, which favoured cartilage regeneration [109]
**Kidney**				
AKI	Human BMMSCs	Ultracentrifugation (100,000× *g*)	Injection/15 µg of total EV protein	Enhanced recovery of injured tubular cells, enhanced tubular cell proliferation, reduced apoptosis [110]
AKI (in vitro)	Human BMMSCs	Ultracentrifugation (100,000× *g*)	Details not provided	Increased renoprotection activity mediated by the transfer of the mRNA for IGF-1 receptor to tubular cells through the MSC-EVs [113]
AKI	Human BMMSCs	Ultracentrifugation (100,000× *g*)	Injection/30 µg of total EV protein	Enhanced tubular epithelial cell proliferation, reduced cell apoptosis [114]
AKI	Mice BMMSCs	Sequential ultracentrifugation (up to 110,000× *g*)	Injection/200 μg of total EV protein in 20 μL PBS	Enhanced tubular epithelial cell proliferation, reduced cell apoptosis [115]
AKI	Human BMMSCs	Ultracentrifugation (100,000× *g*)	Injection/Single dose of 100 μg of total EV protein or multiple doses of 100 μg of total EV protein after cisplatin administration and 50 μg of total EV protein after 2, 6, 10, 14, and 18 days	Upregulated expression of anti-apoptotic genes in cisplatin-treated human tubular epithelial cells and down-regulated expression of cell-apoptotic genes [116]
AKI	Mice BMMSCs	Differential centrifugation and ultracentrifugation (100,000× *g*)	EV or EV-hydrogel injection/80 μg of total EV protein in 15 μL PBS or 80 μg of total EV protein in 15 μL of hydrogel solution	BMMSC-EVs loaded to self-assembling peptide nanofiber hydrogel, showed better EV efficacy and improved renal function [117]
AKI	Human UCMSCs	Differential centrifugation and sucrose gradient centrifugation (100,000× *g*)	Injection/200 μg of total EV protein	Reduced oxidative stress and renal tubular cell apoptosis, increased renal cell proliferation [118]
AKI	Human UCMSCs	Ultracentrifugation (100,000× *g*)	Injection/30 μg of total EV protein	Increased tubular cell proliferation and dedifferentiation [119]
AKI	Human Wharton’s Jelly MSCs	Ultracentrifugation (100,000× *g*)	Injection/100 μg of total EV protein in 1 mL medium 199	Improved renal function by enhancing tubular cell proliferation and reduced inflammation and apoptosis via mitochondrial fission [120,121]
AKI	Human glomerular MSCs	Ultracentrifugation (100,000× *g*)	Injection/EVs derived from 1 × 10^5^ cells in 120 μL PBS ((T-CD133^+^-EVs: 480 × 10^6^/mouse; MSC-EVs: 400 × 10^6^/mouse; MSC-EV-float: 400 × 10^6^/mouse; Fibroblasts-EVs: 230 × 10^6^/mouse)	Enhanced renal recovery [122]
AKI	Human liver MSCs	Ultracentrifugation (100,000× *g*)	Injection/EVs derived from 3.5 × 10^5^ or 10 × 10^5^ MSCs	Enhanced renal function through increased tubular cell proliferation and inhibition of apoptosis [123]
CDK	Human urinary MSCs	Ultracentrifugation (100,000× *g*) followed by sucrose gradient ultracentrifugation (100,000× *g*)	Injection/100 μg of total EV protein in 200 μL PBS	Reduced CKD progression by inhibiting podocyte apoptosis and promoting vascular regeneration and cell survival [125]
CDK	Rat urinary MSCs	Total exosome isolation kit (Life Technologies)	Injection/5.3 × 10^7^ particles in 200 μL PBS	Improved renal morphology through the anti-apoptotic behaviour of tubular epithelial cells [126]
CDK	Human BMMSCs and human liver MSCs	Ultracentrifugation (100,000× *g*)	Injection/1 × 10^10^ particles for each injection once a week for 4 weeks (5 injections)	Reduced CDK progression through miRNAs capable of down-regulating profibrotic genes [127]
CDK	Human liver MSCs	Sucrose gradient ultracentrifugation (350,000× *g*) and ultracentrifugation (100,000× *g*)	Injection/1 × 10^10^ particles in 1 mL PBS for each injection once a week for 4 weeks	Reduced CDK progression through miRNAs capable of down-regulating profibrotic genes [128]
CDK	Mice BMMSC	Ultracentrifugation (100,000× *g*)	Injection/30 μg of total EV protein (3 doses)	Protected renal injury via microRNA-dependent repairing [129,130]
CDK	Human BMMSCs	ExoQuick-TC ULTRA (EQULTRA-20TC-1, SBI)	Injection/4 × 10^7^ particles in 2 μL PBS	Improved renal function by repairing the damage to apical and basolateral membranes and mitochondria of kidney proximal tubules [131]
Grade III-IV CKD (clinical trial)	Human UCMSCs	Ultracentrifugation (100,000× *g*)	Injection/100 μg of total EV protein per kg per dose (2 doses)	Reduced the progression of CDK [132]
**Liver**				
Carbon tetrachloride (CCl4)-induced liver injury	Human embryonic MSCs	Tangential flow filtration and chromatography	Injection/0.4 μg of total EV protein in 100 μL PBS	Increased hepatocyte proliferation and reduced apoptosis [133]
Hepatic ischemia-reperfusion injury	Human iPSCs	Differential centrifugation and ultrafiltration	Injection/600 μg of total EV protein in 400 μL PBS	Enhanced hepatic regeneration via inhibition of apoptosis of hepatic cells, suppression of inflammatory and attenuation of the oxidative stress response [134]
Hepatic ischemia-reperfusion injury	Human iPSCs	ExoQuick Exosome Precipitation Solution (SBI Systems Biosciences)	Injection/2.5 × 10^12^ particles in 500 μL PBS	Increased hepatocyte proliferation in vitro and in vivo in a dose-dependent manner [135]
Hepatic ischemia-reperfusion injury	Human UCMSCs	Ultracentrifugation (100,000× *g*)	Injection/10 mg of total EV protein per kg	Reduced infiltration of neutrophils and oxidative stress in hepatic tissue [139]
Chronic hepatic fibrosis	Human embryonic MSCs	Differential centrifugation and ultracentrifugation (100,000× *g*)	EV-hydrogel injection/350 μg of total EV protein in 400 μL PEG hydrogel solution	Sustained release of MSC-EVs from PEG hydrogels protected against hepatic failure [140]
**Muscle**				
Cardiotoxin-induced muscle injury	Human BMMSCs	Sequential ultracentrifugation (110,000× *g*)	Injection/details not provided	Enhanced muscle regeneration through increased myogenesis and angiogenesis [141]
Cardiotoxin-induced muscle injury	Human amniotic fluid MSCs	Ultracentrifugation (200,000× *g*)	Injection/details not provided	Enhanced muscle regeneration through regulation of inflammation and angiogenesis [142]
Tibialis anterior muscle damage	Human ADMSCs	Ultracentrifugation (200,000× *g*)	Injection/EVs derived from 1 × 10^6^ cells	Enhanced muscle regeneration through the synergistic effect of EVs and soluble proteins [143]
Muscle degeneration associated to torn rotator cuffs	Human ADMSCs	Differential centrifugation and ultracentrifugation (100,000× *g*)	Injection/1 × 10^11^ particles in 20 μL PBS	Enhanced myofiber regeneration and biomechanical properties of muscles in rotator cuffs [144]
Pubococcygeus muscle injury	Human urine-derived MSCs	Differential centrifugation and ultracentrifugation (100,000× *g*)	Injection/1 × 10^10^ particles in 1 mL PBS	Enhanced muscle regeneration through the activation, proliferation, and differentiation of muscle satellite cells [145]
Muscle damage associated to critical limb ischemia	Human ADMSCs	Ultracentrifugation (100,000× *g*)	Injection/1 × 10^10^ particles intravenously or intramuscularly after intervention, 0.5 × 10^10^ via intramuscular injection after day 1 and 2	Enhanced muscle regeneration/protection through NRG1-mediated signals [146]
**Wounds**				
Fibroblasts from normal and chronic wound patients (in vitro)	Human BMMSCs	Differential centrifugation and ultracentrifugation (100,000× *g*)	0.1, 1, and 10 μg total EV protein in 1 mL PBS	Enhanced proliferation and migration of fibroblasts and tube formation by endothelial cells [149]
Skin wound	Human iPSC	Differential centrifugation and ultracentrifugation (100,000× *g*)	Injection/160 μg total EV protein in 160 μL PBS at wound sites and 40 μg total EV protein in 40 μL PBS at wound beds	Increased collagen synthesis and angiogenesis [150]
Photo-damaged dermal fibroblasts in vitro)	Human ADMSCs	Tangential flow filtration	1 × 10^8^ particles in 1 mL PBS	Increased collagen and elastin synthesis and decreased metalloproteinases activity [151]
Skin wound	Human ADMSCs	Ultrafiltration and ExoQuick-TC kit (System Biosciences)	Injection/200 μg of total EV protein in 200 μL PBS	Enhanced wound healing by regulating the collagen distribution secreted by fibroblasts in the early and late stage of wound healing [152]
Excisional skin wound	Human ADMSCs	Differential centrifugation and ultracentrifugation (100,000× *g*)	EV-gel transplantation/1.9 × 10^8^ particles in hydroxyethyl cellulose aqueous gel	Enhanced wound healing through increased migration and proliferation of keratinocytes and fibroblasts [153]
Ischemic wound	Human ADMSCs	ExoQuick-TC kit (System Biosciences)	Injection/details not provided	Enhanced wound healing through migration and proliferation of dermal fibroblasts [154]
Cutaneous wound	Rabbit ADMSCs and BMMSCs	Ultracentrifugation (100,000× *g*)	Injection/EVs derived from 10 × 10^6^ MSCs	Significant better wound healing upon treatment with ADMSC-EVs [155]
Second-degree burn wound	Human UCMSCs	Differential centrifugation and sucrose gradient ultracentrifugation (100,000× *g*)	Injection/200 μg of total EV protein in 200 μL PBS	Enhanced wound healing through increased fibroblasts proliferation and angiogenesis and reduced skin cell apoptosis [156,157,158]
Skin wound	Human UCMSCs	Differential centrifugation and ultracentrifugation (120,000× *g*)	EV-scaffold transplantation/100 μg of total EV protein in 100 μL PBS	Enhanced wound healing and reduced scar formation through inhibition of myofibroblast differentiation [159]
**Other tissues/organs**				
Injuries in lung cells (in vitro)	Human placenta MSC	Differential centrifugation and ultracentrifugation (100,000× *g*)	6 × 10^5^ particles in 20 µL serum-free media	Enhanced cell migration, reduced oxidative cell stress and inflammation [160]
Influenza virus-induced acute lung injury	Swine BMMSCs	Ultracentrifugation (25,000 rpm)	Intratracheally/EVs produced by 10 × 10^6^ MSCs	Reduced virus shedding, production of proinflammatory cytokines, and influenza virus-induced lung lesions [161]
lung injury induced by haemorrhagic shock and trauma	Human BMMSs	Differential centrifugation and ultracentrifugation (100,000× *g*)	Injection/30 μg of total EV protein in 200 μL PBS	Improved pulmonary vascular permeability through the activation of proteins and pathways linked to cytoskeletal rearrangement [162]
Hyperlipidaemia	Human placenta MSC	Differential centrifugation and ultracentrifugation (100,000× *g*)	EV-scaffold transplantation/100 μg of total EV protein in 1 mL PBS	Reduced calcification of synthetic vascular grafts by immunomodulation and improved vascular function [163]
Neointimal hyperplasia (in vitro)	Human ADMSCs	Differential centrifugation and ultracentrifugation (100,000× *g*)	100 μg of total EV protein in 1 mL PBS	Reduced abnormal proliferation and migration of vascular smooth muscle cell [164]
Hind-limb ischemia	Human UCMSCs	Differential centrifugation and ultracentrifugation (100,000× *g*)	Injection/2 × 10^10^ particles in 1 mL PBS	Enhanced angiogenesis and muscle regeneration, and ischemic limp function [165]
Tracheoesophageal fistulas	Porcine ADMSCs	Ultracentrifugation (100,000× *g*)	EV-gel injection/1.3 × 10^11^ particles in 1 ml of pluronic F127 gel	Enhanced oesophageal fistula healing through targeted delivery of EVs embedded in thermo-responsive hydrogels [166]
Ulcerative colitis	Mouse BMMSCs	Differential centrifugation and ultracentrifugation (100,000× *g*)	Injection/50 μg of total EV protein per day for 7 days	Improved symptoms through stimulating M2 macrophage polarization and negative inflammatory response [167]

**Table 2:** List of clinical trials using MSC-EVs against tissue injuries *.

Tissue Injury/Disease	Condition	Treatment	Trial Phase	Trial ID
Lung injury	Healthy	Aerosol inhalation of allogenic ADMSC-EVs (2 × 10^8^ particles/3 ml or 1 × 10^8^ particles/3 ml)	Phase I	NCT04313647
Lung disease (pneumonia)	Coronavirus disease-19	Aerosol inhalation of allogenic ADMSC-EVs (2 × 10^8^ particles/3 ml) for 5 days	Phase I	NCT04276987
Chronic lung disease	Bronchopulmonary dysplasia	Intravenous infusion of BMMSC-EVs (20 or 60 or 200 pmol phospholid/kg body weight)	Phase I	NCT03857841
Cartilage injury	Osteoarthritis	Osteochondral explants from arthroplasty patients treated with ADMSC-EVs	Phase I	NCT04223622
Brain injury	Acute ischemic stroke	Administration of allogenic MSC-EVs enriched with miR-124 (200 μg total EV protein) via Stereotaxis	Phase II	NCT03384433
Skin injury	Dystrophic Epidermolysis Bullosa	Allogenic BMMSC-EVs locally administrated	Phase II	NCT04173650
Kidney disease	CDK	Injection of allogenic UMMSC-EVS (100 μg of total EV protein per kg per dose)	Phase II/III (completed)	[132]

* Information obtained from https://clinicaltrials.gov/ on 8 April 2020.
